# Human Umbilical Cord Mesenchymal Stem Cells Protect Against Steroid-Induced Osteonecrosis of the Femoral Head Through Hippo Pathway

**DOI:** 10.3390/biomedicines14030727

**Published:** 2026-03-22

**Authors:** Hengte Xing, Wenxiang Cai, Junwen Chen, Hanzhe Xu, Yubiao Zhang, Changheng Zhong, Jianlin Zhou, Hao Peng

**Affiliations:** Department of Orthopedics Surgery, Renmin Hospital of Wuhan University, Wuhan 430060, China; xinghengte@whu.edu.cn (H.X.); caiwx1@whu.edu.cn (W.C.); 2018283020157@whu.edu.cn (J.C.); 2024283020153@whu.edu.cn (H.X.); zhangyubiao@whu.edu.cn (Y.Z.); 18706827939@163.com (C.Z.)

**Keywords:** human umbilical cord mesenchymal stem cells, steroid-induced osteonecrosis of the femoral head, microvascular endothelial cells, ferroptosis, Hippo pathway

## Abstract

**Background:** Glucocorticoids (GCs) are a key pathogenic factor in steroid-induced avascular necrosis of the femoral head (SANFH). GCs can directly damage bone microvascular endothelial cells (BMECs), leading to impaired intraosseous blood supply. Recent studies suggest the Hippo signaling pathway may be involved in the pathogenesis of SANFH; however, its role in vascular endothelial repair and angiogenesis remains unclear. This study aims to investigate the therapeutic effects of human umbilical cord mesenchymal stem cells (hUC-MSCs) on SANFH, with a particular focus on their protective or reparative mechanisms on BMECs. **Methods:** In vivo, a SANFH mouse model is established and divided into NC, MPS, and hUC-MSCs groups, followed by Micro-CT imagin, hematoxylin and eosin (HE) staining and immunohistochemistry (IHC) (*n* = 8 per group). In vitro, BMECs are divided into NC, dexamethasone (Dex), hUC-MSCs, and Fer-1 groups to analyze cellular biological behaviors. Target protein expression is assessed using Western blotting and immunofluorescence microscopy. Ferroptosis-related markers are detected via biochemical assays. Mitochondrial ultrastructural changes are observed using transmission electron microscopy. **Results:** In vivo, the MPS group exhibited significant bone cavitation, sparse trabeculae, and disrupted trabecular architecture in the femoral head. The hUC-MSCs group showed marked improvement in bone microstructure, HE staining showed a significant decrease in the empty lacunae rate in the femoral head, and IHC results revealed markedly increased expression of cluster of differentiation 31 (CD31) and vascular endothelial growth factor (VEGF). In vitro, Dex stimulation suppressed BMECs proliferation. In Dex-treated cells, levels of intracellular reactive oxygen species (ROS), lipid peroxides, ferrous ion (Fe^2+^), malondialdehyde (MDA), acyl-CoA synthetase long chain family member 4 (ACSL4) and nicotinamide adenine dinucleotide phosphate oxidase 4 (NOX4) were all increased, while expression of glutathione (GSH) and glutathione Peroxidase 4 (GPX4) was reduced. Transmission electron microscopy revealed plasma membrane rupture and reduction or loss of mitochondrial cristae. Furthermore, Dex promoted Hippo-mediated phosphorylation of Yes-associated protein (YAP)/Transcriptional coactivator with PDZ-binding motif (TAZ), upregulated NOX4 expression, and suppressed CD31 and VEGF expression. Following hUC-MSCs treatment, BMECs demonstrated enhanced proliferation, migration, and tube-forming capacity. Cellular GSH and GPX4 levels increased, antioxidant capacity was restored, peroxide accumulation decreased, and cells were protected from ferroptosis-effects comparable to those in the Fer-1 group. Additionally, hUC-MSCs inhibited YAP/TAZ phosphorylation and promoted elevated expression of CD31 and VEGF. **Conclusions:** These findings suggest that hUC-MSCs may attenuate Dex-induced ferroptosis in BMECs, enhance BMEC migration and angiogenesis, and improve femoral head microstructure in SANFH through modulation of the Hippo-YAP/TAZ signaling pathway. This study provides novel insights into the therapeutic potential of hUC-MSCs for SANFH.

## 1. Introduction

Steroid-induced avascular necrosis of the femoral head (SANFH) is a common and difficult-to-treat disease in orthopedics. Following the prolonged or high-dose use of glucocorticoids (GCs), it leads to impaired blood microcirculation within the femoral head. This results in progressive necrosis of the local bone tissue, bone marrow, and osteocytes, destruction of the bone microstructure, and ultimately leads to femoral head collapse and hip joint dysfunction [1]. Currently, total hip arthroplasty (THA) is an effective and definitive treatment for this condition. Since the age of onset of SANFH does not show significant specificity, an epidemiological study indicates that there are over 8 million patients with osteonecrosis of the femoral head (ONFH) in China among individuals aged 15 and above. However, artificial hip joints have a limited lifespan. Undergoing THA too early inevitably necessitates revision surgery, imposing significant physical trauma and financial burden on both patients and their families. Therefore, younger patients often prefer hip-preserving treatments [2]. Simultaneously, GCs are widely used in clinical practice and are irreplaceable in the treatment of various diseases. Therefore, it is imperative to conduct more comprehensive and in-depth exploration into the pathogenesis of SANFH, in order to provide effective targeted therapeutic strategies for its prevention and treatment.

The etiology of SANFH is complex and diverse. Currently, impaired microvascular circulation within the femoral head and abnormal lipid metabolism are considered major risk factors. Consequently, pharmacological interventions for early-stage SANFH primarily include anticoagulants, vasodilators, lipid-lowering agents, and bisphosphonates. However, their efficacy is limited and insufficient to effectively delay disease progression. With the advancement of modern biomedical technologies, an increasing number of studies are focusing on the use of mesenchymal stem cells (MSCs) for treating ONFH [3]. MSCs are a type of adult stem cell characterized by strong proliferative capacity and the ability to differentiate into multiple lineages. They are diverse in type and abundant in source. Among them, human umbilical cord mesenchymal stem cells (hUC-MSCs) stand out due to their advantages such as low immunogenicity, high in vitro expansion capability, easy accessibility, and minimal ethical controversy [4]. Studies have shown that hUC-MSCs may promote the repair of local blood vessels and bone tissue following injury—thereby delaying the progression of SANFH—by participating in the regulation of the local microenvironment, oxidative stress, and immune responses, as well as by modulating osteocyte death, angiogenesis osteogenesis coupling, and the homeostasis of osteogenic adipogenic differentiation [5,6,7]. Based on the proposed hypothesis of “angiogenic-osteogenic coupling,” damage to and dysfunction of bone microvascular endothelial cells (BMECs) are believed to induce impaired blood supply within the femoral head, which is considered a critical factor influencing the progression of SANFH [8].

Ferroptosis, as a novel form of regulated cell death, has attracted significant attention in recent years. It is characterized by the lethal accumulation of iron-dependent lipid peroxides (LPO) within cells, resulting from dysregulation of cellular metabolism and redox mechanisms. Glutathione (GSH) and glutathione peroxidase 4 (GPX4) are crucial antioxidants that protect cells against ferroptosis [9]. Studies have observed the occurrence of ferroptosis during the progression of SANFH, though most research has focused on osteoblasts, osteoclasts, and bone marrow mesenchymal stem cells, uncovering some of the underlying mechanisms of ferroptosis in these cells. However, the interaction between glucocorticoid-induced BMEC injury and ferroptosis, as well as its specific mechanisms, remains unclear [10,11,12].

The Hippo signaling pathway is a highly conserved kinase cascade pathway. Recent studies have shown that it is widely involved in regulating various mechanisms in SANFH, including osteogenesis, chondrogenesis, and lipid metabolism in bone cells, thereby influencing bone metabolism as well as bone repair and remodeling [13,14,15]. Furthermore, the Hippo signaling pathway also plays a significant role in vascular remodeling. YAP/TAZ are key components in angiogenesis, involved in regulating the proliferation, migration, and spatial arrangement of endothelial cells, and also help prevent hemorrhage in developing blood vessels [16]. Additionally, studies have shown that ferroptosis can be regulated through the Hippo signaling pathway in retinal capillary endothelial cells, pulmonary vascular endothelial cell lines, and pericytes [17,18,19].

A reduction or disruption of intra-femoral head blood supply constitutes a major pathological feature of SONFH. While the Hippo signaling pathway plays a crucial role in vascular remodeling and the maintenance of vascular integrity across multiple diseases, its specific impact on BMECs in the context of SONFH remains unexplored. Thus, targeting this pathway could represent a novel therapeutic strategy for the early intervention of SONFH.

This study utilized a methylprednisolone-induced early SONFH mouse model to assess the protective effects of hUC-MSCs on femoral head bone microstructure and microvessels. Mechanistically, using a Dex-induced BMECs injury model in vitro, we report for the first time that hUC-MSCs suppress ferroptosis in mouse BMECs through the Hippo signaling pathway. This anti-ferroptotic action preserves endothelial function, facilitates microvascular formation, and ultimately enhances local blood perfusion, offering protection against SONFH.

## 2. Materials and Methods

### 2.1. In Vivo Animal Experiments

The experiments were performed at the Animal Experiment Center of the Renmin Hospital, Wuhan University. All animal procedures followed the guidelines for laboratory animal care and adhered to the ARRIVE guidelines. Ethical approval for the experiments was obtained from the Laboratory Animal Ethical and Welfare Committee of the Renmin Hospital of Wuhan University (WDRM20220506A).

In this study, healthy male C57BL/6 mice aged 2–3 months (20–24 g) were purchased from Hunan SJA Laboratory Animal Co., Ltd., Changsha, China. To control for biological variability, mice of the same age and weight from the same breeding batch were randomly assigned to each group. All mice were acclimatized under specific pathogen-free conditions for 1 week, and those with body weights outside the range of 18–26 g or exhibiting poor health status were excluded. A total of 24 male C57BL/6 mice were randomly divided into three groups (*n* = 8 per group) using a random number table method: the NC group, MPS group, and hUC-MSCs group. SONFH mouse model was established by a combination of intraperitoneal (i.p.) injection of lipopolysaccharide (LPS) and intramuscular (i.m.) injection of methylprednisolone (MPS) into the gluteus maximus muscle. On days 1–2, the MPS and hUC-MSCs groups received i.p. injections of LPS (20 µg/kg) at 24 h intervals. From days 3–14, the MPS and hUC-MSCs groups received i.m. injections of MPS (20 mg/kg) into the left gluteal muscle daily at 24 h intervals. Starting from week 3, the frequency of MPS injections was reduced to every other day for 2 consecutive weeks. Simultaneously, the hUC-MSCs group received tail vein injections of a 0.9%NaCl suspension containing hUC-MSCs (5 × 10^5^ cells per mouse) beginning on day 3. The injections were administered twice weekly until the end of week 2, and then once weekly for 2 consecutive weeks starting from week 3. Meanwhile, both the control group and the model group received an equivalent volume of 0.9%NaCl injection at the same time points as the hUC-MSCs group. After the final injection, all mice were observed for 4 weeks, then euthanized, and samples were collected for further analysis. All subsequent outcome assessments were performed by two independent investigators who were blinded to the experimental groups.

### 2.2. Micro-CT Examination and Analysis

Micro-CT analysis was performed on the femoral head tissues of 24 mice using a scanner (SkyScan 1276 micro-CT system, Bruker, Kontich, Belgium). The parameters were set as follows: peak energy, U = 85 kV, I = 200 μA. The scanning slice gap was 10.5 μm, and the intermediate frequency resolution was 1024 × 1024. The data were acquired and reconstructed with the NRecon software (v1.7.4.2), and the 3D reconstruction was carried out using CTvox (v3.3). The measured morphometric parameters included trabecular separation (Tb.Sp, mm), trabecular number (Tb.N, 1/mm), bone volume per tissue volume (BV/TV, %), and trabecular thickness (Tb.Th, mm).

### 2.3. Immunohistochemistry of Bone Tissue

Bone tissue specimens were fixed in 4% paraformaldehyde for 24–48 h and then decalcified in 10% ethylenediaminetetraacetic acid (EDTA) solution with daily changes until complete decalcification was confirmed by needle penetration. Following dehydration through a graded ethanol series, clearing in xylene, and paraffin embedding, consecutive sections of 4–5 μm thickness were prepared. Paraffin sections were deparaffinized through a gradient of ethanol to water, followed by antigen retrieval and allowed to cool naturally. After rinsing with PBS, the sections were outlined with an immobilized wax pen and blocked with BSA at room temperature for 30 min. Primary antibodies (CD31, 1:800 dilution, Novus Biologicals, Centennial, CO, USA; VEGF, 1:200 dilution, Santa Cruz, CA, USA) were then applied and incubated at 4 °C overnight, secondary antibodies were added and incubated for 1 h at room temperature in the dark. The nuclei were counterstained with DAPI for 10 min. After a final PBS wash, the sections were mounted with an antifade mounting medium. Fluorescent images were captured and recorded under an optical microscope (Olympus, Hachioji-shi, Tokyo, Japan).

### 2.4. Preparation and Characterization of hUC-MSCs

The hUC-MSCs were purchased from Shenzhen Wingor Biotechnology Co., Ltd., China [20]. Moreover, the cells used in the experiments were obtained from 3–6 generations of the same batch of cells. The cells were cultured using the DMEM/F12 (HyClone, Logan, UT, USA) supplemented with 10% FBS (Serapro, USA) and a 1% penicillin mixture (Biosharp, Beijing, China). The cell culture was maintained at 37 °C with 5% CO_2_. The medium was refreshed every 2–3 days when the cells reached approximately 80–90% passage.

### 2.5. Cell Culture

Primary mouse BMECs (QS-M290, Keycell Biotechnology Co., Wuhan, China) were purchased from Wuhan Keycell Biotechnology Co., Ltd, Wuhan, China. Cells from the same batch between passages 3 and 6 were used for the experiments. The cells were cultured in Endothelial Cell Medium (ECM, ScienCell, Carlsbad, CA, USA) supplemented with 5% fetal bovine serum (FBS, ScienCell, Carlsbad, CA, USA), 1% endothelial cell growth supplement (ECGS, ScienCell, Carlsbad, CA, USA), and 1% penicillin/streptomycin solution (P/S, ScienCell, Carlsbad, CA, USA). A non-contact co-culture system was employed for the experiments. In the Transwell co-culture system (0.4 μm pore size, Corning, NY, USA) using 6-, 24-, and 96-well plates, hUC-MSCs and BMECs were seeded in the upper and lower chambers at specific ratios, respectively, and maintained in ECM. The experimental groups were as follows, NC group (no intervention), Dex group (induced with Dex), hUC-MSCs group (induced with Dex and co-cultured with hUC-MSCs for 24 h), Fer-1 group (induced with Dex and 1μM Fer-1 for 24 h). All in vitro experiments were repeated at least three times independently (biological replicates) using cells from different passages (P3-P6). Within each experiment, each assay was performed in triplicate (technical replicates).

### 2.6. Cell Counting Kit-8 (CCK8)

The impact of Dex on BMECs viability was assessed using the Cell Counting Kit-8 (CCK-8, Keycell, Wuhan, China). Cells were seeded into 96-well cell culture plates and incubated overnight at 37 °C in a 5% CO_2_ atmosphere. They were then treated with dexamethasone (Dex, MCE, Monmouth Junction, NJ, USA) at the following concentrations (1, 10, 100, and 1000 μM) for 24 h. Thereafter, 10 μL of CCK-8 reagent was added to each well, followed by incubation at 37 °C for 2 h. The absorbance of each well was measured at 450 nm using a microplate reader. Based on the results, an appropriate concentration of Dex was selected for subsequent experiments. CCK-8 was also used to evaluate cellular viability under different co-culture ratios. As previously described, BMECs were seeded in the lower chamber of 24-well plates, while hUC-MSCs were seeded in the upper chamber at corresponding ratios. The co-culture ratios between upper and lower chambers were as follows: 0:1 (blank control group), 0:1, 1:1, 2:1, 4:1, 8:1, and 10:1. Finally, absorbance was measured at 450 nm using a microplate reader. An optimal co-culture ratio was selected for follow-up experiments.

### 2.7. Immunofluorescence

BMECs were seeded on sterile coverslips placed in culture plates and allowed to reach 70–80% confluence before treatment. The cells on coverslips were fixed with ice-cold 4% paraformaldehyde for 15 min at 4 °C, followed by washing with PBS. Permeabilization was performed using 0.5% Triton X-100 (diluted in 1× PBS) for 5 min at room temperature. Then the samples were blocked with diluted goat serum for 30 min at room temperature. Primary antibodies (CD31, 1:100 dilution, Bioss, China; vWF, 1:100 dilution, Proteintech, Wuhan, China) were then applied and incubated overnight at 4 °C, secondary antibodies (Cy3-conjugated goat anti-rabbit IgG, 1:100 dilution, BOSTER, Pleasanton, CA, USA) were added and incubated for 1 h at 37 °C in a humidified chamber. DAPI was added and incubated for 5 min in the dark. Anti-fade mounting medium was applied, and the coverslips were air-dried before being imaged under a fluorescence microscope.

### 2.8. Hematoxylin–Eosin (HE) Staining

Femoral head tissues were fixed in 10% paraformaldehyde overnight and then decalcified in 10% EDTA for 2 months; the decalcification solution was changed every 3 days. Following decalcification, the specimens were dehydrated using an automatic dehydrator, embedded in paraffin, and sectioned at a thickness of 5 µm. The sections were then deparaffinized in xylene for 10 min, rehydrated through a graded alcohol series, stained with hematoxylin for 5 min, differentiated with 5% acetic acid for 5 min, and counterstained with eosin for 3 min. After dehydration through graded alcohol, the sections were cleared and mounted for microscopic observation. The rate of bone cavitation was analyzed under a high-power field.

After the mice were sacrificed for femoral head collection, their major organs were harvested and immediately fixed. Following a series of procedures including dehydration, embedding, and clearing, the samples were sectioned, dried in an oven, deparaffinized, rehydrated, and rinsed with distilled water before staining. After dehydration, the sections were sealed with coverslips and observed and photographed under a microscope.

### 2.9. Wound Scratch Assay

BMECs were seeded in the lower chamber, while hUC-MSCs were seeded in the upper chamber at designated ratios. When the cells reached approximately 90% confluence, they were subjected to group-specific interventions to assess cell migratory capacity. The groups were treated for 24 h as follows: NC group: Upper chamber with hUC-MSC culture medium; lower chamber with BMECs. Dex group: Upper chamber with hUC-MSC culture medium; lower chamber with BMECs treated with a specified concentration of Dex. hUC-MSCs group: Upper chamber with hUC-MSCs at a specific ratio; lower chamber with BMECs treated with Dex. Fer-1 group: BMECs treated with Dex and 1 μM Fer-1. All subsequent experiments adhered to these group assignments. A uniform scratch wound was created in the confluent cell monolayer using a 200 µL pipette tip held vertically. The dislodged cells were removed by gently washing with PBS, and serum-free medium was added to the wells. Images of the scratch were captured immediately (0 h). The plate was then placed in a cell culture incubator (37 °C, 5% CO_2_) for continued culture. Subsequent images were taken at 12 h and 24 h after scratching. The migration rate was quantified by analyzing the wound area using Image J (v1.0).

### 2.10. Matrigel Invasion Assay

BMECs from each group were harvested by trypsinization and resuspended in serum-free medium at a density of 2 × 10^5^ cells/mL. Matrigel (Corning, NY, USA) was thawed at 4 °C overnight. Transwell inserts, 24-well plates, and pipette tips were pre-cooled at −20 °C overnight. All subsequent steps were performed on ice. Each well of the 24-well plate was filled with pre-chilled ECM medium. Matrigel was applied to the bottom of each insert, followed by incubation at 37 °C for 1 h to facilitate gel formation. Then, cell suspensions were added to the upper chambers. After incubation, the cells were fixed for 1 h, stained with 0.5% crystal violet solution for 20 min at room temperature. The results were observed and documented under a microscope. Image J software was used for quantitative image analysis.

### 2.11. Tube Formation Assay

BMECs from each experimental group were harvested by trypsinization and resuspended in serum-free medium to a density of 1.5 × 10^5^ cells/mL. Matrigel was thawed in advance at 4 °C. A 12-well culture plate and pipette tips were pre-chilled at −20 °C overnight. Then, 100 µL of Matrigel was added to each well and incubated for 1 h to allow polymerization into a gel matrix. Subsequently, 1 mL of the cell suspension was added into each well, followed by incubation for 6 h. The results were observed and recorded under a microscope. Image J software was used for quantitative image analysis.

### 2.12. Transmission Electron Microscopy

Cell clusters were fixed with 2.5% glutaraldehyde (Alfa Aesar, Ward Hill, MA, USA) for 2–4 h, followed by three 15-min rinses with PBS. Subsequently, the samples were post-fixed with 1% osmium tetroxide (Ted Pella, Redding, CA, USA) for 2 h at room temperature and rinsed three times with PBS. Dehydration was performed using a graded ethanol series. The samples were then infiltrated overnight with a 1:1 mixture of acetone and EPON 812 resin (SPI, West Chester, PA, USA), embedded in pure EPON 812 resin, and polymerized at 60 °C for 48 h. Ultrathin sections were prepared and stained with uranyl acetate and lead citrate (SPI, West Chester, PA, USA). After air-drying overnight at room temperature, the sections were observed and imaged using a transmission electron microscope.

### 2.13. Western Blot

Total protein was extracted from BMECs in each experimental group using RIPA lysis buffer, and the protein concentration was quantified using a BCA assay kit (G3522-2, GCBIO Technologies, Guangzhou, China). After electrophoresis and membrane transfer, the PVDF membrane was blocked with 5% skim milk in TBST for 2 h at room temperature on a shaker. The membrane was then incubated with specific primary antibodies (GPX4, 1:1000 dilution, Bioss, Beijing, China; ACSL4, 1:1000 dilution, Affinity Biosciences, Jiangsu, China; NOX4, 1:2000 dilution, Proteintech, Wuhan, China; YAP, p-YAP, TAZ, 1:1000 dilution, Cell Signaling Technology, Danvers, MA, USA; p-TAZ, 1:1000 dilution, Affinity Biosciences, Jiangsu, China; CD31, 1:2000 dilution, Abcam, Cambridge, UK; VEGF, 1:200 dilution, Santa Cruz, Dallas, TX, USA; GAPDH, 1:50,000 dilution, Proteintech, Wuhan, China) diluted in TBST at 4 °C overnight. The following day, the membrane was washed with TBST and incubated with corresponding HRP-conjugated secondary antibodies diluted in TBST for 2 h at room temperature with shaking. After thorough washing with TBST, the membrane was treated with ECL working solution for signal development and exposure. The obtained images were subjected to gray value quantitative analysis using IPP 6.0 software.

### 2.14. Determination of Malondialdehyde (MDA) and Glutathione (GSH)

The levels of MDA and GSH in BMEC lysates were evaluated using an MDA assay kit (A003-1-1, Nanjing Jiancheng Bioengineering Institute, Nanjing, China) and a GSH assay kit (A006-2-1, Nanjing Jiancheng Bioengineering Institute, Nanjing, China), respectively. BMECs were cultured in vitro, digested, and collected to prepare protein lysates. The protein concentration was determined using a BCA protein assay kit (G3522-2, GCBIO Technologies, Guangzhou, China). The MDA and GSH levels were then assessed and normalized according to the protein concentration. All subsequent procedures were strictly performed in accordance with the manufacturers’ instructions. For MDA, the absorbance was measured at 532 nm using a microplate reader. For GSH, the absorbance was measured at 405 nm. The contents of MDA and GSH were calculated based on their respective standard formulas.

### 2.15. Determination of Intracellular Ferrous Ion (Fe^2+^)

Intracellular Fe^2+^ levels were measured using a Ferrous Iron Content Assay Kit (BC5415, Solarbio, Beijing, China) strictly according to the manufacturer’s protocol. Briefly, BMECs in good growth condition were homogenized in the provided extraction buffer, and the supernatant was collected after centrifugation. The absorbance was measured at 593 nm. A standard curve was plotted based on the instructions, and the Fe^2+^ concentration was calculated accordingly.

### 2.16. Determination of ROS

Intracellular ROS levels were detected using a Reactive Oxygen Species Assay Kit (Beyotime Biotechnology, Shanghai, China) according to the manufacturer’s instructions. Briefly, the culture medium was aspirated from BMECs in the lower chamber, and the cells were gently washed with PBS. Then, the diluted DCFH-DA working solution was added, and the cells were incubated in the culture incubator for 20 min. After washing with serum-free medium, Hoechst 33342 (Yeasen Biotechnology, Shanghai, China) working solution was added, and the cells were further incubated for 10 min. Following another wash with serum-free medium, the cells were observed and imaged under a fluorescence microscope. Optical density analysis was performed on immunofluorescence images using IPP 6.0 software.

### 2.17. Determination of C11

LPO levels were measured using the C11 BODIPY 581/591 Cell Detection Kit (Thermofisher, Waltham, MA, USA). As described previously (see Section 2.5), BMECs were seeded in 24-well plates and grown to 80% confluence. The medium was aspirated from BMECs in the lower chamber, washed with pre-warmed PBS, and the diluted BODIPY 581/591 C11 working solution (prepared in serum-free medium) was added. The cells were incubated in the incubator in the dark for 30 min. After incubation, the working solution was removed, and the cells were washed with serum-free medium. Hoechst 33342 working solution was added, and incubation continued in the dark for 10 min. Following a final wash with serum-free medium, oxidized and reduced state images were observed and recorded under a fluorescence microscope. Optical density analysis was performed on immunofluorescence images using IPP 6.0 software.

### 2.18. Flow Cytometry

After 14 days of hUC-MSCs intervention, mouse orbital blood was collected. A total of 100 μL of whole blood was placed into an EDTA anticoagulant tube. Red blood cells were lysed in flow tubes and incubated in the dark for 10 min until the solution became transparent. Lysis was then terminated, and cells were resuspended and counted. After staining cell surface antigens with fluorescent antibodies, samples were analyzed by flow cytometry to evaluate changes in T and B cell subsets in peripheral blood. Data were collected and quantitatively analyzed using FlowJo software (v10). Antibodies used for flow cytometry included anti-CD4 (AC0062, Beyotime, Shanghai, China), anti-CD8 (AC063, Beyotime, Shanghai, China), and anti-CD19 (AC0516, Beyotime, Shanghai, China).

### 2.19. Statistical Analysis

All data are presented as the mean ± standard deviation (SD) from at least three independent experiments. Statistical analysis was performed using Graph Pad Prism 9.0. For normally distributed data, the *t*-test, one-way ANOVA, and Tukey’s test were used. A value of *p* < 0.05 was considered statistically significant.

## 3. Results

### 3.1. Safety Evaluation of Multiple Injections of Heterologous hUC-MSCs in Mice

**Figure 1:**
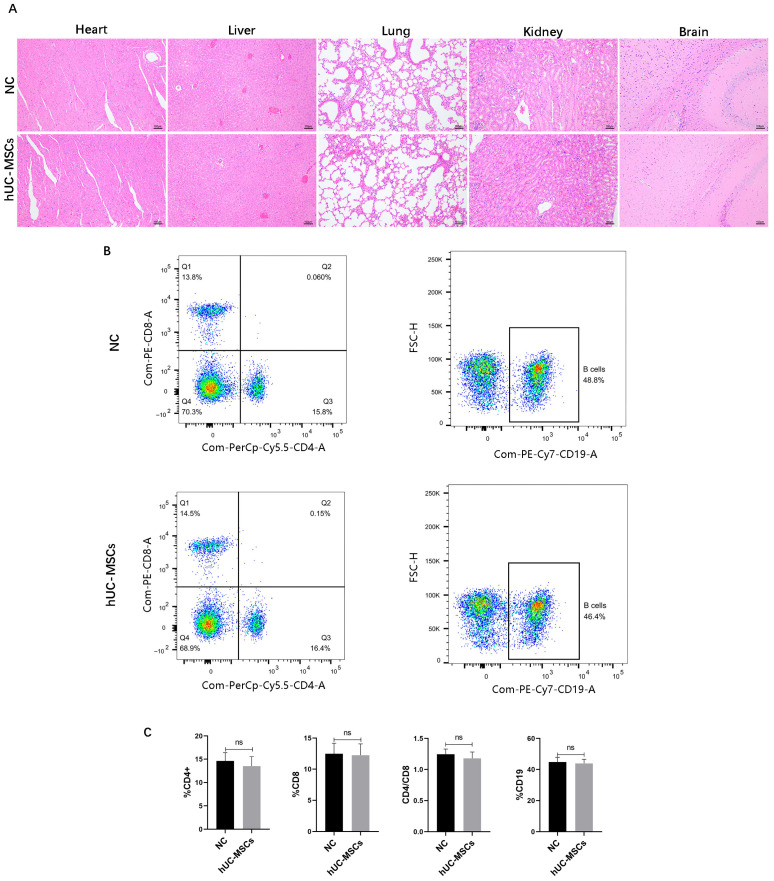
Safety evaluation of multiple injections of heterologous hUC-MSCs in Mice. HE staining of vital organs including the heart, liver, spleen, lung, kidney and brain (**A**). (scale bar = 100 μm). Representative histograms of T-cell subsets and B-cell subsets of NC and hUC-MSCs group in flow cytometry (**B**). Analysis of changes in the ratios of CD4, CD8, CD4/CD8, and CD19 (**C**). The data are presented as the means ± SD (*n* = 4 per group) (ns for not significant, *p* > 0.05).

To evaluate the safety profile of repeated hUC-MSCs administrations in mice, histopathological analyses were performed on major organs, including the heart, liver, lungs, kidneys, and brain. HE staining (Figure 1A) revealed no significant pathological abnormalities or inflammatory cell infiltration in the hUC-MSC-treated group compared to the normal control NC group. Furthermore, to assess potential immunomodulatory effects, peripheral blood lymphocytes were analyzed by flow cytometry. The proportions of T-cell subsets (CD4^+^ and CD8^+^) and B-cells (CD19^+^) showed no statistically significant differences between the NC and hUC-MSCs groups (Figure 1B,C). These findings suggest that repeated intravenous administration of hUC-MSCs does not elicit significant adverse immune responses or organ toxicity in mice under the conditions of this study.

### 3.2. hUC-MSCs Ameliorated Femoral Head Microarchitecture in a GCs-Induced SANFH Mouse Model

**Figure 2:**
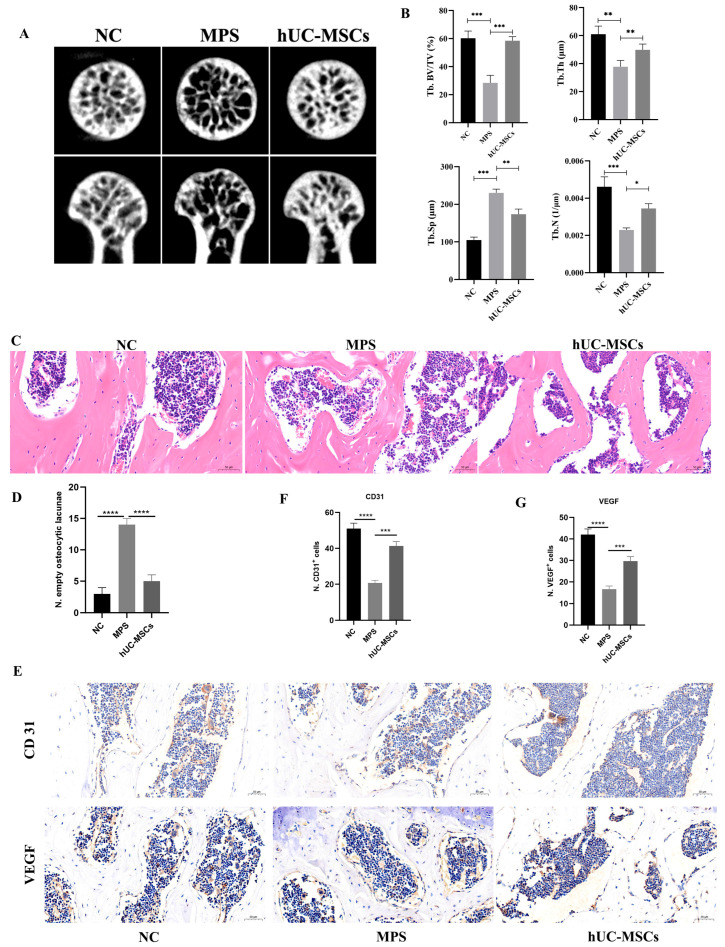
hUC-MSCs Infusion ameliorates femoral head microstructure in SONFH mouse model. Micro-CT images of mouse femoral heads in each group (**A**). Quantitative analysis of BV/TV, Tb/Th, Tb/N, Tb.Sp in each group (**B**). HE staining of mouse femoral head tissue (**C**) (scale bar = 50 μm), quantitative analysis of the number of empty lacunae in cells (**D**). Immunohistochemistry of CD 31 and VEGF (**E**) (scale bar = 50 μm), quantitative analysis of the expression levels of CD31 (**F**) and VEGF (**G**). The data are presented as the means ± SD (*n* = 8 per group). * *p* < 0.05, ** *p* < 0.01, *** *p* < 0.001, **** *p* < 0.0001, compared with the MPS group.

Micro CT analysis revealed significant bone cavitation, disorganized architecture, and sparse trabeculae in the femoral heads of MPS-treated mice compared to the NC group. In contrast, the hUC-MSC-treated group exhibited increased BV/TV, Tb.Th, and Tb.N, along with decreased Tb.Sp, indicating more complete and well arranged trabecular structure (Figure 2A,B). These data suggest that hUC-MSCs effectively improve bone microarchitecture in SANFH. HE staining and immunohistochemistry for CD31 and VEGF were performed to evaluate intraosseous angiogenesis (Figure 2C). The hUC MSC-treated group showed markedly elevated expression of CD31 and VEGF within the femoral head (Figure 2D), suggesting enhanced microvascular formation that may contribute to delayed SANFH progression.

### 3.3. hUC-MSCs Counteracted the Inhibitory Effect of GCs on the Proliferative Activity of BMECs

**Figure 3:**
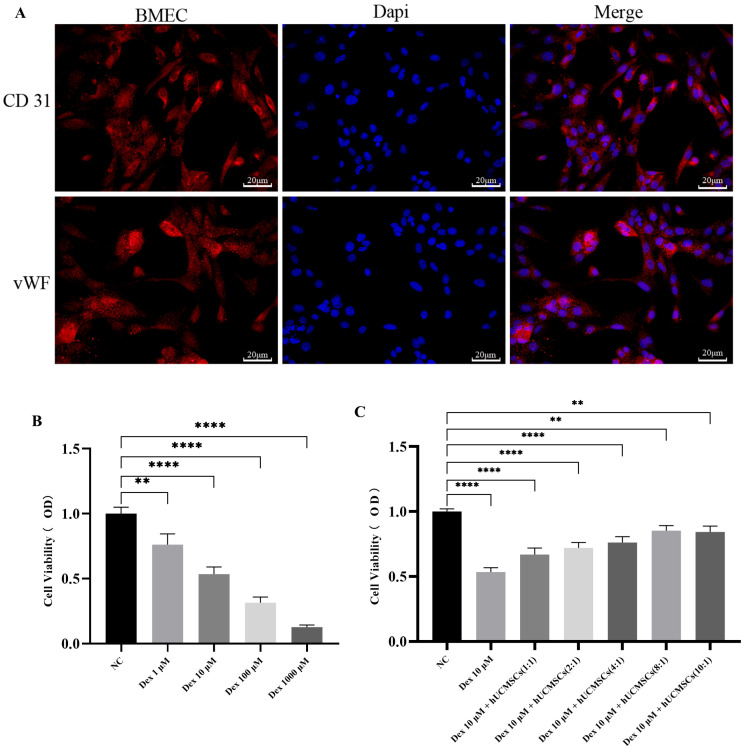
Identification of BMECs and effects of DEX and hUC-MSCs on BMEC viability. Identification of BMECs by immunofluorescence staining for CD31 and vWF (**A**) (scale bar = 20 μm). Effects of different Dex concentrations (1, 10, 100, 1000 μM) on BMEC viability were determined by CCK-8 (**B**). Effect of different hUC-MSCs co-culture ratio (1:1, 2:1, 4:1, 8:1, 10:1) on BMEC viability at 10 μM Dex were determined by CCK-8 (**C**). Data are presented as mean ± SD from technical replicates (*n* = 3). ** *p* < 0.01, **** *p* < 0.0001, compared with the NC group.

Recent studies have demonstrated a close association between BMEC injury and the pathogenesis of SANFH. To investigate the underlying mechanisms, primary mouse BMECs were isolated and cultured. Morphological observation under light microscopy revealed that the cells predominantly exhibited a short rod shaped, spindle shaped, or polygonal morphology, while appearing oval during growth and confluence. Immunofluorescence staining confirmed strong positive expression of CD31 and vWF (Figure 3A), confirming the endothelial identity of the isolated cells. Subsequent experiments were performed to evaluate the effects of Dex and hUC-MSCs on BMEC viability. CCK 8 assays demonstrated that Dex significantly suppressed BMEC viability in a concentration dependent manner, with a marked reduction observed at 100 μM Dex (Figure 3B). Further CCK 8 assays evaluating the effect of hUC-MSCs on BMEC viability under various co culture ratios revealed that a hUC-MSCs: BMECs ratio of 8:1 significantly rescued Dex-induced suppression of cell viability (Figure 3C). Based on these results, subsequent in vitro experiments were conducted using 10 μM Dex as the injury condition and a hUC-MSCs:BMECs co culture ratio of 8:1 as the intervention condition.

### 3.4. hUC-MSCs Promote the Migration, Invasion, and Tube Formation of BMECs

**Figure 4:**
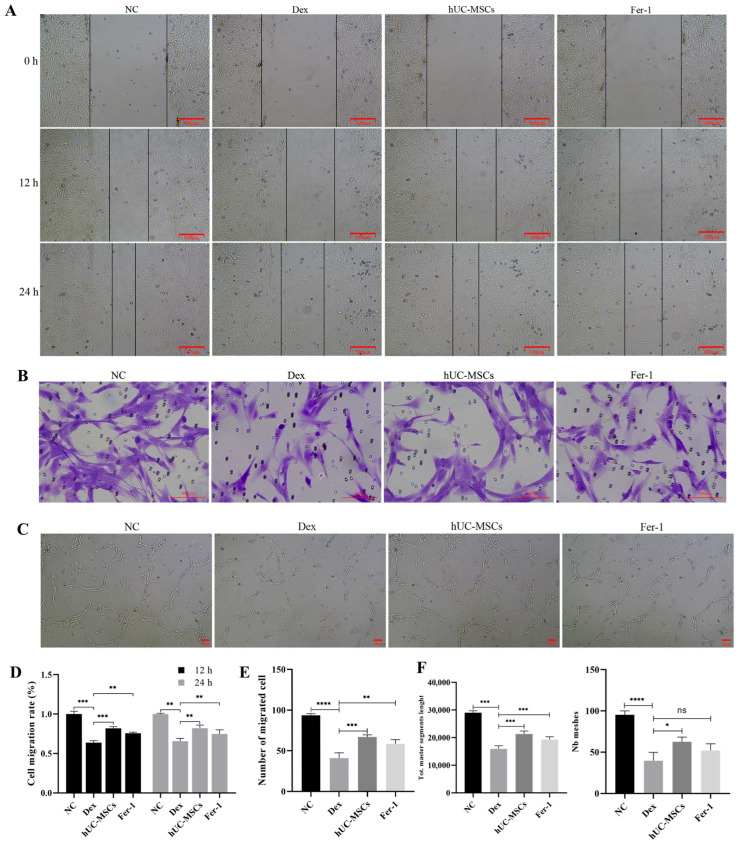
Effects of Huc-MSCs on BMECs Proliferation, Migration, and Angiogenic tube of formation. Under conditions of 10 μM Dex and an 8:1 hUC-MSCs: BMEC co-culture ratio, wound healing after Dex treatment (0, 12, 24 h) (**A**,**D**) (scale bar = 100 μm). Cell migration measured by Transwell assay (**B**,**E**) (scale bar = 100 μm). BMEC tube formation capability was determined (**C**,**F**) (scale bar = 100 μm). Data are presented as mean ± SD from technical replicates (*n* = 3) * *p* < 0.05, ** *p* < 0.01, *** *p* < 0.001, **** *p* < 0.0001, ns for not significant, compared with the Dex group.

As previously described, BMECs play a crucial role in intraosseous microvascular formation. To evaluate the angiogenic capacity of BMECs in vitro, this study assessed their migration, invasion, and tube formation abilities. The results demonstrated that, compared to the NC group, Dex significantly inhibited BMEC migration (Figure 4A,D) and invasion (Figure 4B,E), as well as suppressed tube formation (Figure 4C,F). However, when BMECs were co cultured with hUC-MSCs, the hUC-MSC-treated group exhibited markedly enhanced proliferation, invasion, and tube formation capabilities (Figure 4).

### 3.5. hUC-MSCs Attenuated GCs-Induced Iron Overload and Oxidative Stress in BMECs

**Figure 5:**
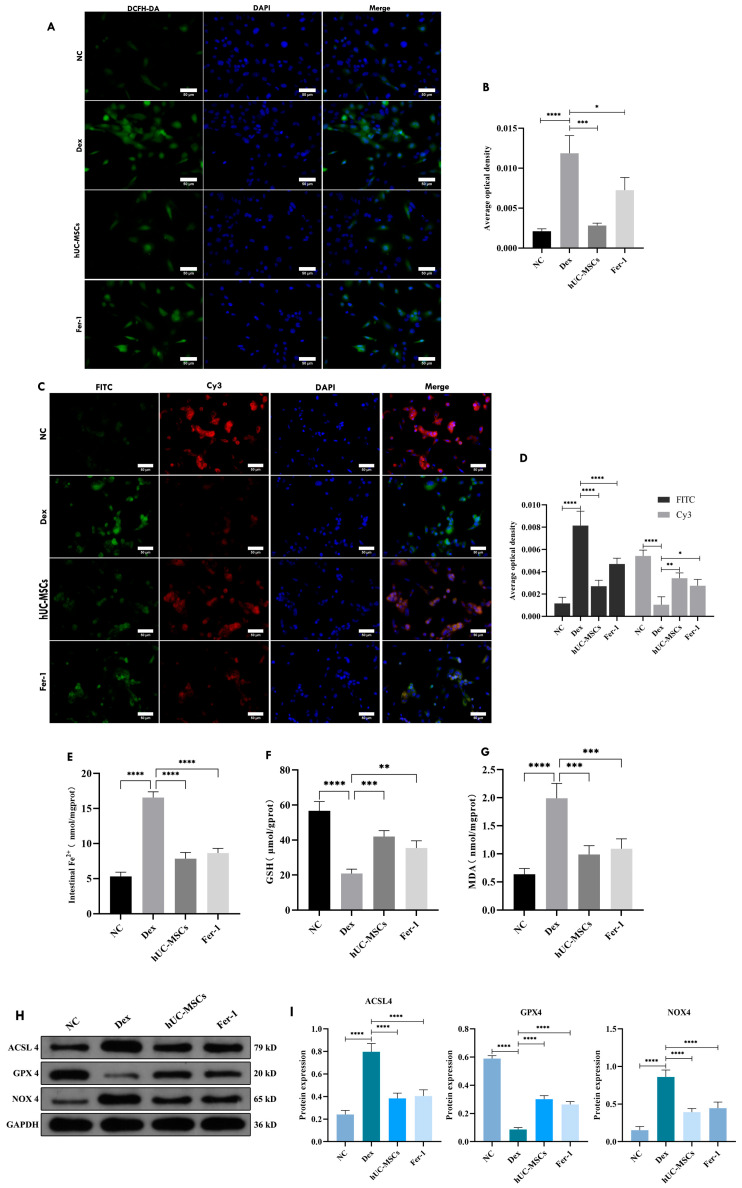
HUCMSCs suppress ferroptosis in BMECs induced by Dex treatment. ROS (**A**,**B**) and LPO (**C**,**D**) levels in BMECs in each group (scale bar = 50 μm). Fe^2+^ (**E**), GSH (**F**) and MDA (**G**) content in BMECs. Expression of ferroptosis-related proteins (ACSL4, GPX4, NCOA4) by Western blot (**H**,**I**). Data are presented as mean ± SD from technical replicates (*n* = 3). * *p* < 0.05, ** *p* < 0.01, *** *p* < 0.001, **** *p* < 0.0001, compared with the Dex group.

To investigate whether ferroptosis occurs in BMECs following dex intervention, this study examined the expression changes in peroxides, iron ions, and ferroptosis-related proteins. Immunofluorescence results demonstrated that, compared to the NC group, dex-treated cells exhibited significantly increased intracellular ROS and lipid peroxide levels (Figure 5A–D). Biochemical assays further revealed elevated iron ion and MDA levels in BMECs (Figure 5E,G), while the antioxidant GSH was markedly reduced (Figure 5F), indicating oxidative stress, abnormal peroxide accumulation, and diminished antioxidant capacity. These alterations were reversed by hUC-MSCs intervention, which significantly decreased intracellular ROS, lipid peroxides, Fe^2+^, and MDA, while restoring GSH levels and enhancing antioxidant activity. Compared to the NC group, Dex treatment upregulated ACSL4 and NOX4 and downregulated GPX4. In contrast, both the hUC-MSCs and Fer-1 groups exhibited increased GPX4 expression and reduced ACSL4 and NOX4 levels compared to the Dex group (Figure 5H,I), suggesting that hUC-MSCs attenuate intracellular peroxide accumulation and iron overload, thereby suppressing oxidative stress.

**Figure 6:**
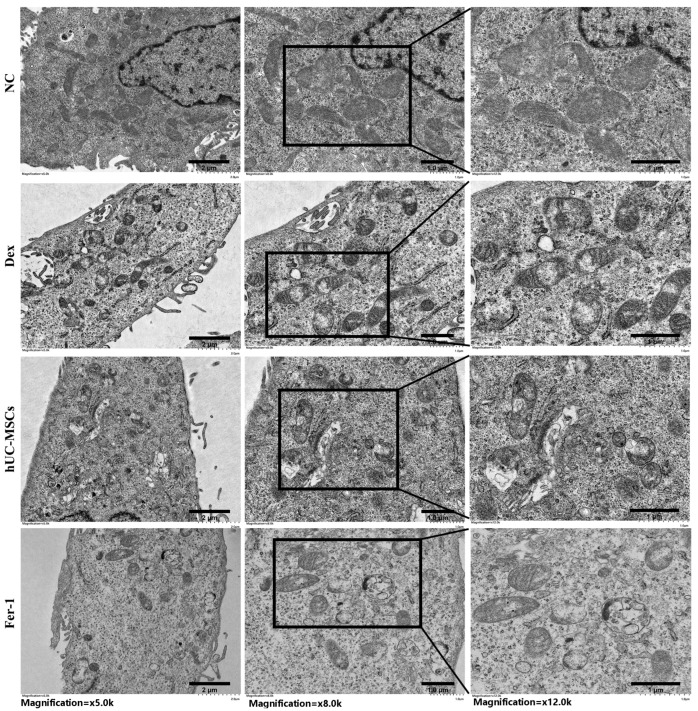
Inhibition of mitochondrial deformation by hUC-MSCs. Mitochondrial morphology in BMECs was observed by TEM at original magnifications of ×5.0 k, ×8.0 k, and ×12.0 k. Data are presented as mean ± SD from technical replicates (*n* = 3).

Furthermore, mitochondrial morphological alterations observed by electron microscopy are considered a hallmark of ferroptosis. In the Dex group, BMECs displayed ruptured mitochondrial outer membranes and reduced or absent cristae (Figure 6). Collectively, these findings demonstrate that Dex induces ferroptosis in BMECs, while hUC-MSCs mitigate mitochondrial damage, reduce peroxide and iron accumulation, and likely inhibit ferroptosis in BMECs.

### 3.6. hUC-MSCs Alleviated GCs-Induced Ferroptosis in BMECs via the Hippo Signaling Pathway

**Figure 7:**
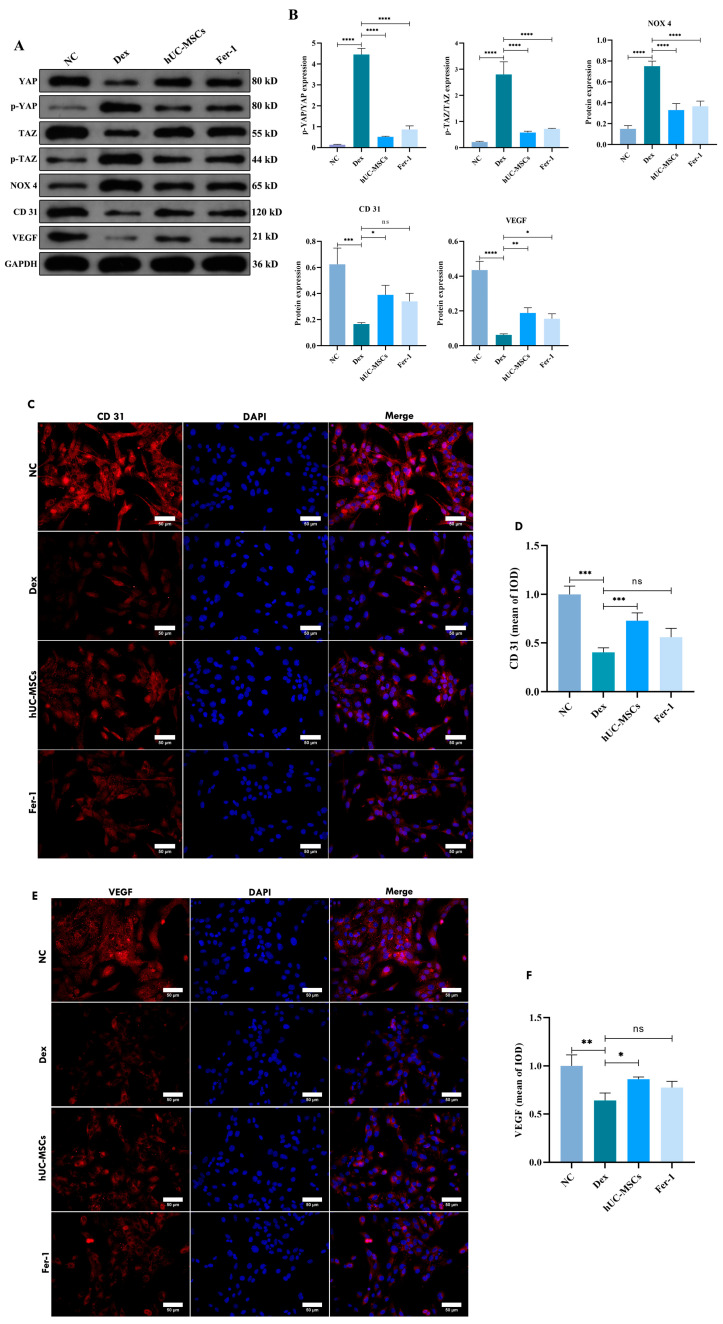
hUC-MSCs inhibit Dex-induced ferroptosis in BMECs via the Hippo-YAP-TAZ signaling pathway. The protein expression levels of YAP, *p*-YAP, TAZ, *p*-TAZ, NOX4, CD31, and VEGF in BMECs from each group were detected by Western blot (**A**,**B**). Immunofluorescence staining and quantitative analysis of CD31 (**C**,**D**) and VEGF (**E**,**F**) expression in BMECs from each group (scale bar = 50 μm). Data are presented as mean ± SD from technical replicates (*n* = 3). * *p* < 0.05, ** *p* < 0.01, *** *p* < 0.001, **** *p* < 0.0001, ns for not significant, compared with the Dex group.

The Hippo pathway is recognized as closely associated with angiogenesis and vascular development, while BMECs are critical for intraosseous microvascular formation in the femoral head. To investigate the underlying mechanisms of Dex-induced ferroptosis in BMECs and the protective effects of hUC-MSCs, this study examined the expression levels of Hippo pathway-related proteins in BMECs. The results revealed that Dex promoted excessive phosphorylation of YAP/TAZ via the Hippo pathway, significantly upregulating the ferroptosis-related protein NOX4 (Figure 7A,B). Concurrently, expression of CD31 and VEGF in BMECs was markedly suppressed (Figure 7A,B), suggesting that Dex may induce ferroptosis in BMECs through the Hippo pathway and inhibit the expression of angiogenesis-related proteins, thereby impairing local microvascular formation and exacerbating microcirculatory dysfunction. In contrast, both hUC-MSCs and Fer 1 significantly inhibited the phosphorylation of the YAP/TAZ pathway and reduced NOX4 production. Compared to the Dex group, the hUC MSC group exhibited enhanced protein expression of CD31 and VEGF (Figure 7A,B). Immunofluorescence analysis further confirmed increased expression of CD31 and VEGF in BMECs following hUC-MSC treatment (Figure 7C–F). In summary, hUC-MSCs exert a protective effect on BMECs, potentially through modulation of the Hippo pathway. This mechanism may safeguard BMECs against Dex-induced ferroptosis, promote microvascular formation within the femoral head, improve local blood supply, and consequently delay the progression of SANFH.

## 4. Discussion

Impaired intraosseous blood supply has long been recognized as one of the primary etiological factors in the development of SANFH. BMECs are highly active endocrine cells that primarily line the luminal surface of intraosseous microvessels. They participate in regulating multiple physiological processes and coordinate bone–vessel crosstalk, with their close coupling being essential for skeletal development [21]. Studies have reported that excessive GCs use can directly damage vascular endothelium, induce microvascular thrombosis and microcirculatory dysfunction, ultimately leading to osteonecrosis of the femoral head [22]. Therefore, focusing on vascular endothelial repair or promoting angiogenesis within the femoral head may represent a critical therapeutic strategy for SANFH.

Currently, stem cell therapy has garnered increasing attention due to its homing effect and paracrine capabilities. Stem cells can migrate and release various cytokines, participating in the regulation of multiple immune responses in the body. Under different conditions, they can be induced to differentiate into diverse tissues such as bone, cartilage, and muscle, thereby promoting the repair and healing of damaged tissues. This approach demonstrates promising efficacy in the treatment of bone and joint diseases [23,24,25,26]. It has been reported that the clinical outcomes of stem cell implantation are similar to those of core decompression alone for ONFH. Notably, when stem cell therapy is combined with core decompression, it can effectively delay the progression of ONFH and reduce the rate of conversion to THA following decompression [27]. hUC-MSCs are a type of stem cell present in the human body. Due to their easy accessibility and low immunogenicity, they stand out among various stem cell sources. Clinical studies on the use of human umbilical cord mesenchymal stem cells (hUC-MSCs) for osteonecrosis of the femoral head (ONFH) remain limited. In a pioneering study, Chen et al. conducted a 3-year follow-up of nine patients with ONFH who received intra-arterial infusion of hUC-MSCs via the femoral artery. Radiographic assessment revealed a significantly lower rate of necrotic lesion progression post-treatment compared to preoperative baselines, along with improved local bone microarchitecture. These findings suggest that intra-arterial hUC-MSC infusion is a feasible and relatively safe therapeutic strategy for ONFH. Nevertheless, the underlying pathophysiological and molecular mechanisms responsible for these observed effects were not elucidated in that study [28]. Additionally, a Phase I clinical trial investigating stem cell-based therapy for SONFH has been reported, in which osteoblasts derived from hUC-MSCs were directly implanted into the necrotic lesions. A short-term follow-up of 12 weeks was conducted primarily to assess adverse events, evaluate the safety profile of the cell therapy, and determine the optimal dosage and treatment timing. The trial protocol also includes a long-term follow-up period of 5 years, during which imaging examinations are used to monitor the progression of femoral head necrosis before and after treatment. The clinical outcomes are currently still under observation [29]. Therefore, to promote clinical research on hUC-MSCs in the treatment of SONFH, the underlying mechanism warrants further investigation. Recent studies have further demonstrated that hUC-MSCs possess strong pro-angiogenic capabilities. By inhibiting the activation of the NLRP3 inflammasome, hUC-MSCs alleviate vascular inflammation and endothelial cell pyroptosis in Kawasaki disease, significantly improve endothelial dysfunction, and promote the proliferation of umbilical vein endothelial cells and vascular formation [30]. Based on the “angiogenic-osteogenic coupling” hypothesis in SANFH, we established a mouse model of SANFH to observe the effects of hUC-MSCs. Before the formal experiments were conducted, we performed a preliminary evaluation of the safety of repeated allogeneic stem cell injections for therapeutic purposes, an often overlooked consideration in current stem cell-based therapies. In this study, HE staining of major mouse organs, combined with the analysis of lymphocyte phenotypes in peripheral blood, revealed no significant immune response or organ toxicity following tail vein injection of hUC-MSCs in mice. These findings demonstrate that hUC-MSC administration was safe under the conditions of this study.

And the results showed that hUC-MSCs significantly improved the bone microstructure of the femoral head in the SANFH mouse model, which aligns with our research group’s previous findings in a rabbit model of SANFH. This suggests that hUC-MSCs may promote osteogenesis and protect the femoral head [31,32]. Meanwhile, IHC results of the femoral head in this study revealed that hUC-MSCs also promoted the expression of angiogenesis-related markers CD31 and VEGF within the femoral head. In vitro experiments further demonstrated that hUC-MSCs significantly improved the proliferation, migration, and tube forming capacities of BMECs inhibited by Dex, suggesting that in SANFH, hUC-MSCs may promote intra femoral microvascular regeneration, enhance blood supply, and thereby facilitate bone repair and delay disease progression. Additionally, this study further explored the mechanisms underlying Dex-induced injury in BMECs. Under electron microscopy, it was observed that Dex-induced rupture of the mitochondrial outer membrane, reduction in cristae, and a significant increase in intracellular peroxide and iron ion levels in BMECs. The expression of key ferroptosis-related proteins ACSL4 and NOX4 was markedly elevated, while GPX4 expression decreased, indicating a weakened cellular antioxidant capacity. These findings suggest that Dex can induce ferroptosis in BMECs. In contrast, under hUC MSC treatment, the above phenomena in BMECs were significantly ameliorated: ferroptosis was suppressed, and oxidative stress was alleviated. This effect was similar to the results observed after intervening with Fer 1 (a potent ferroptosis inhibitor) on BMECs. These results indicate that hUC-MSCs can significantly inhibit Dex-induced ferroptosis in BMECs.

Vascular remodeling is a fundamental pathological process in disease progression, involving various growth factors and signaling pathways. The Hippo signaling pathway is one of the key pathways that participates in regulating vascular endothelial cell function, inducing vascular remodeling, maintaining vascular integrity, and thereby protecting the organism [33]. For example, Zhang et al. [34] found that in a mouse model of collagen-induced arthritis, restoring the activity of the Hippo YAP signaling pathway inhibited vascular endothelial cell activation and angiogenesis in the synovium, while also reducing the inflammatory response in the synovial tissue, thereby protecting the joints. Liu et al. [35] demonstrated in a hyperglycemia-induced retinal capillary injury model that YAP1, a downstream effector of the Hippo pathway, can regulate ferroptosis and inflammation in retinal capillary endothelial cells, thereby delaying disease progression. Additionally, studies have shown that the Hippo signaling pathway regulates apoptosis in human umbilical vein endothelial cells and modulates the formation of atherosclerotic plaques [36,37]. Furthermore, recent studies have reported that the Hippo signaling pathway may also play a significant role in glucocorticoid-induced osteonecrosis of the femoral head (ONFH). Kuang et al. [38] found that exosomal miR 365a 5p derived from hUC-MSCs regulates osteocyte apoptosis via the Hippo signaling pathway and promotes osteogenesis in SANFH. Li et al. [39] discovered that the Hippo signaling pathway can influence the osteogenic adipogenic balance in bone marrow mesenchymal stem cells, thereby delaying the progression of ONFH. Current research has primarily focused on bone marrow stem cells, osteoblasts, and osteoclasts, aiming to promote osteogenesis in bone cells, improve bone quality, and protect the femoral head. Therefore, we hypothesize that the Hippo signaling pathway may also play a significant role in the process of vascular repair and remodeling within the femoral head in SANFH. This study found that Dex can induce ferroptosis in BMECs and inhibit angiogenesis through Hippo-mediated overexpression of YAP/TAZ phosphorylation. In contrast, hUC-MSCs can suppress YAP/TAZ activation, downregulate the expression of key ferroptosis-related proteins such as NOX4 and ACSL4 in cells, reduce ferroptosis in BMECs, and simultaneously increase the expression of angiogenesis-related proteins CD31 and VEGF, thereby promoting vascular formation by BMECs. These findings demonstrate that in SANFH, the Hippo signaling pathway is involved in regulating the functional repair of BMECs and vascular remodeling.

Although this study provides evidence that hUC-MSCs can stimulate vascular remodeling within the femoral head in SANFH, certain limitations exist. First, in the in vivo experiments using a SONFH mouse model, the assessment of hUC-MSC effects on femoral head vasculature was limited to static histological markers (CD31 and VEGF). Functional vascular evaluations, such as angiography, quantitative analysis of vessel density, and blood perfusion measurements, were not performed. Therefore, although the presence of vascular structures was confirmed, whether these vessels were functionally normal remains undetermined. Second, although the Hippo signaling pathway is one of the key pathways in vascular remodeling during disease, other pathways such as TGF β1/Smads and VEGF also play crucial roles in angiogenesis and vascular injury repair—and both are closely related to the Hippo signaling pathway [33]. This study did not demonstrate the influence of hUC-MSCs on the interactions among these three pathways. Finally, recent studies have demonstrated that hUC-MSCs can secrete a variety of cytokines, growth factors, chemokines, and extracellular vesicles, influencing cellular functions through paracrine mechanisms. We have not yet clarified whether hUC-MSCs also participate in regulating ferroptosis in BMECs via such paracrine modes. Further research is needed to explore this, which could provide new insights into the mechanisms of stem cell therapy for SANFH. The safety of stem cell injection is of paramount importance. Although some studies have indicated that repeated allogeneic injections of mesenchymal stem cells can exert therapeutic effects without exacerbating rejection, and other research has demonstrated that even under immune stimulation, allogeneic hUC-MSCs maintain low immunogenicity in humanized mouse models, concerns remain [40,41]. For instance, some scholars have reported adverse events following intrathecal injection of MSCs in patients with musculoskeletal diseases, albeit not particularly severe [42]. Therefore, attention must be paid to the safety of allogeneic MSC injections, and further evaluation in larger and more diverse preclinical models and clinical trials is essential. This remains a major challenge in stem cell therapy, as current research has yet to confirm that every MSC injection.

In summary, this study demonstrates that hUC-MSCs can inhibit Dex-induced ferroptosis in BMECs in vitro and promote the migratory and tube forming biological behaviors of endothelial cells. The underlying mechanism may involve hUC-MSCs suppressing the Dex-induced activation of the Hippo YAP/TAZ signaling pathway. Concurrently, in vivo experiments also revealed that hUC-MSCs significantly improved both bone quality and local microvascular repair in the femoral heads of SANFH mice.

## Data Availability

All data are presented in the manuscript.
